# 

**Published:** 1854-01

**Authors:** 


### INDEX.

A

Abel, F. A. and C. L. Bloxam's handbook of chemistry, bibl. notice 415
Abortion, hemorrhage in 569
Amputation, Dr. D. Gilbert's case of, above the shoulder joint 593
Adhesive plaster, Dr. Neill's use of, in treatment of fractures of patella 1
Abscesses, scrofulous, of the cerebellum, case 11
Atlee, W. L., prize essay on fibrous tumors of the uterus, bibl. notice of 27
Armor, Dr. Samuel G., prize essay bibl. notice 40
Aneurismal tumors, both carotids tied 60
Alcoholic liquors, use and abuse, bibl. notice 40
Alcohol, behavior of, in the animal organism 90
Ashwell, Dr. Samuel, on diminution, &c. of uterine tumors 247
Aneurism, statistics of treatment of, by compression 250
Anæsthesia, employment of Hardy's instrument for local 255
Anæsthesia, failure of local 634
Anæsthesia by cold, unsuccessful 683
Albumen, in the urine, after scarlatina, by H. Bence Jones 255
Anatomy, comparative, by C. H. Siebold &c., H. Stannius, bibl. notice 308
American Medical Association, proceedings of, at St. Louis, 1854 361
Ayre, Dr., on treatment of cholera 434
Arteries, elasticity of, as a cause of animal heat 434
Anasarca, treated by decoction of onions 550
Auscultation and Percussion, Dr. Skoda's treatise on, bibl. notice 608, 665
Ayres, Dr. E. D., on treatment of malignant pustule 633
Arsenic, eaters of 685
Atlee on M. Bernard's lectures on the blood, bibl. notice of 750

B

Baillarger on expansion of surface of brain, &c. 122
Barbadoes, cholera in 510
Bennett, Dr. J. Hughes, on pathology and treatment of tuberculosis, bibl. notice 235
Bennett, Dr. J. Henry, practical treatise on inflammation of the uterus, &c. bibl. notice 356
Buckler, Dr. Thos. H., on the etiology, &c., of rheumatic pneumonia, bibl. notice 35
Bright's disease, microscopic examination of case of 72
Butter, removal of rancidity 189
Brathwaite's Retrospect, bibl. notice 238
Burnett, Dr Waldo J., translation of Siebold, &c., comparative anatomy, bibl. notice 308
Billing, Dr. Archibald's, first principles of medicine, bibl. notice 357
Brinton, Dr., on a new method of preserving anatomical specimens, &c. 398
Blood, changes in, from cod-liver and cocoa-nut oil 57

Brain, Dr. R. Wagner on elementary organization of the 601
Bushman, Dr. S., principles of animal and vegetable physiology, bibl. notice 627
Blockley Hospital, opening of 628
Bremner, John, on comparative pathology of post-partum hemorrage 636
Bickersteth's cure of hypertrophy of the nose 637
Berlin, report of Dr. Graefe's clinic 660
Braun on eclampsia 689
Buck, Dr. Gurdon, case of successful operation for vesico-vaginal fistula 697
Bernard's lectures on the blood, by Dr. Walter F. Atlee, bibl. notice of 750

C

Carpenter, Dr. Wm. B., on the use and abuse of alcoholic liquors, bibl. notice 40
Carpenter, D. Wm. B., principles of comparative physiology, bibl. notice 675
Carpenter, Dr. B. D., cases of tetanus treated by ice 61
Calomel, S. Jackson on, in diseases of children 129
Carson's edition of Pereira's Materia Medica, bibl. notice 552
Cartwright, Dr., on dysentery among negroes 630
Coffee, chemico-physiological effects of 19, 98
Cholera in Europe, remarks on 42
" in Barbadoes 510
" Sargent's notes of a few cases 529
Cholera on board emigrant ships, 42
" Ayre's treatment of 434
Cholera, etiological anomalies in 575
" in London 680
" different modes of treatment of 699
Chloroform, accidents from 42
Chloroform, death from 111, 185
Chemistry and metallurgy, by A. S. Piggott, M. D., bibl. notice 108
College of Physicians of Phila. election of delegates to American Medical Association 184
Chamberlayne, Dr. Lewis, death of 185
Cleveland, W. F.'s case of phthisis with pneumo-thorax, &c. 190
Compression, statistics of treatment of aneurisms 250
Cellulose, Virchow's discovery of, in the brain 267, 333
Chemistry, elementary, theoretical and practical, by G. Fownes, bibl. notice 354
Chlorides, diagnostic value of the absence of, in the urine 382
Consumption, Clinical Lectures on, by T. Thompson, M. D.. bibl. notice 410
Cyanosis, Dr. T. W. Shaw's case of 459
Cesarean section, translated from the Spanish 548
Cesarean section, two cases of successful 587
Constipation, Prof. Trousseau on 571
Cod-liver oil, effects on the blood 574

D.

Darrach, Dr. Jas., case of tubercular peritonitis, &c. 321
Death of Dr. Hester, of New Orleans, 43; of Dr. S. McClellan, 112; Dr. J. Ester Cooke, 112; Dr. A. A. Giltenan, 112; Dr. L. Chamberlayne, 185; Dr. Shattuck, 240; Dr. Howard, 241; Dr. Swett, Dr. V. Mott, Jr., Dr. Carter P. Johnson, Drs. P. H. Wildman, F. W. Schley, S. N. Harris, T. M. Ellis, C. H. Welles, 680, 681
Deaths from chloroform 111, 185
Ducheck, Dr., on the behavior of alcohol 90
Diseases of the eye, treatise on, bibl. notice 105
Detachment of fibrinous deposits from interior of the heart 113
Dieffenbach's operation for ununited fracture 446
Dispensatory. of the U. S., Wood & Baches', 10th edition, bibl. notice 561
Dysentery, Dr. A. Flint's clinical report on, bibl. notice 563
Dysentery among negroes, Dr. Cartwright on 630

Diarrhœa in phthisis, treatment of 631
Diabetes, John Horsley's new test for sugar in 684
Double uterus, twins and placenta previa, Hohl's case of 691
Dictionary of medical terminology, dental surgery, and the collateral sciences, by Chapin A. Harris, M. D., bibl. notice 757

E

Earle, Dr. Pliny, on institutions for the insane, bibl. notice 564
Earle, Dr. Pliny, examination of the practice of blood-letting in mental diseases, bibl. notice 564
Eclampsia in pregnancy, Braun on 689
Emphysema, Dr. J. F. Meigs' case of subcutaneous 7
Election of Dr. Socrates Maupin 43
" of Dr. R. J. Breckenridge 43
Expansion of surface of brain, in connexion with its development 122
Editorial on the American Medical Association 316
Editorial on the meeting of the American Medical Association 357
Editorial on the meeting of the Pennsylvania Medical Society 431
Electricity, Dr. Littell on, as a cause of disease 385
Elasticity of arteries, as a cause of animal heat 434
Erichsen's science and art of of surgery, bibl. notice 477
Epilepsy and other affections of nervous system, by Radcliffe, bibl. notice 500
Endocarditis, Lebert's view on 565
Exhaustion from effects of heat Dr. H. S. Swift on 567
Eye, treatment of wounds of 632
Elkoplasty or Anaplasty, Dr. F. N. Hamilton on, bibl. notice 673
Ether inhalation, psychical effects of, by Dr. Moreton Stille 730

F

Falck, on effects of clysters of of pure water 249
Female doctors 702
Fever hole, a London 638
Flint, Dr. Joshua B., report on surgery, bibl. notice 38
Flint, Dr. Austin, clinical report on dysentery and chronic pleurisy, bibl. notice 563
Foreign bodies in stomach and duodenum 123
Fungus of the upper jaw, Dr. J. Neill's case of removal 195
Forceps, in two cases of labor, by F. H. Ramsbotham, M. D 252
Filtration of the air, by H. Schroder and Von Dusch 327
Fownes, Geo., elementary chemistry, &c. bibl. notice 354
First principles of medicine, by A. Billing, M. D., bibl. notice 357
Fuller, Dr. on rheumatism, &c., bibl. notice 401
Fractures, Dieffenbach's operation for ununited 446
Formulary, Dr. Thomas Griffith's universal, bibl. notice 510

G

Gastrotomy for rupture of the uterus 577,694
Gedding's, Dr. E., case of extirpation of the uterus for inversion 692
Gibbs, Dr. O. C., on treatment of inflammatory rheumatism 87
Gibbs, Dr. D., on case of chronic pneumonia, &c. 265
Gilbert, Dr. D., case of amputation above the shoulder-joint 593
Gout, Dr. Caspar Morris on death from acute attack of 641
Gilman, Dr. John T., a case of gastrotomy for rupture of the uterus 694

H

Hamilton, Dr. F. H. on elkoplasty &c., bibl. notice 673
Handy, Dr. Washington R., text book on anatomy, bibl. notice 36
Handbook of chemistry and bibl. notice 415
Hartshorne, Dr. Ed., case of penetrating wound of intestine, &c. 645
Harvard College, donation to 43

Harris, Dr. Chapin A. dictionary of medical terminology, dental surgery, and the collateral sciences, bibl. notice of 755
Hartshorne's remarks on the case of a dentist convicted of violating a patient while under the influence of ether inhalation 705
Hester, death of Dr., 43
Hemorrhage, case of, from inversion of the uterus 45
Hemorrhage in abortions 569
Hemorrhage, post partum, comparative pathology of 636
Hemorrhage, suppression in cancer uteri 639
Homœopathy, its tenets and tendencies by Dr. J. S. Simpson, bibl. notice 165
Homœopathy fairly represented by Dr. Wm. Henderson, bibl. notice 165
Henderson, Dr. W., on homœopathy, and bibl. notice 165
Heart and the aorta, diseases of, by Wm. Stokes, bibl. notice 181
Heart, malformation of the, Peacock's lectures on 440
Human anatomy, Dr. T. G. Richardson's elements of, bibl. notice 237
Hutchison, Dr. Joseph C., on the use of common salt in intermittent fever 241
Hudson, Dr. D. M., case of monstrosity 326
Hospital therapeutics, short notices of 444
Hospital cases, by Dr. John Neill 449
Hughes, Dr. H. M., clinical introduction to practice of auscultation, bibl. notice 608, 665
Hypertrophy of the nose, cure of 637
Hilgard, Dr. T. C., brief report of clinics of Berlin 660
Horsley, John, new test for sugar in diabetes 684
Hohl's case of double uterus, twins and placenta previa 691

I

Inflammatory rheumatism, treatment of 87
Intestine, Dr. John Neill's case of gun shot wound of 161
Intestine, penetrating wound of 645
Intermittent fever, Dr. Wm. Pepper's treatment by quinidine 564
Intermittent fever 574
Inversion of the uterus, Gedding's extirpation of the organ 692
Iodide of zinc, employment of 59

J

Jackson, Dr. Saml. (of Northumberland,) on calomel in diseases in children 129
Joynes, Dr. Levin S., appointment of 43
Jewell, Dr. Wilson, on mortality of Philadelphia for Oct. Nov. and Dec. 77; for Jan. Feb. and March 272; April, May and June 465; July, Aug. and Sept. 651.
Johnston, Dr. Christopher, on homologous tubera of the liver 193
Jones, Dr. N. Bence, on albumen in the urine, after scarlatina 255

K

Kennerly, Dr. S., case of quadruple birth 693
Kirkpatrick, Prof. Jas. A.,
Meteorological Observations for
" November 64
" December 127
" January, 1854 192
" February 256
" March 320
" April 384
" May 448
" June 512
" July 576
" August 640
" September 704
" October 758
" for the year 1853 128
Kirkes, Dr. Wm. Senhouse, on detachment of fibrinous deposits from interior of heart 113
Kolliker's manual of human microscopical anatomy, &c. bibl. notice 617

L

La Roche, Dr. R., on pneumonia, bibl. notice 220, 282
Lawrence, W., treatise on disease of the eye, bibl. notice 105
Lehman, Julius, on the chemico-physiological effects of coffee 19, 98
Lewis, Dr. F. W.'s case of scrofulous abscesses of cerebellum 11
Lewis, Dr. F. W.'s cases of obscure diseases of the lungs 257
Lewis, Dr. Samuel on connection of tubercular matter with inflammation 146
Liver, Dr. C. Johnston on homologous tubera of 193
Liver, Lereboullet on intimate structure of 204
Lereboullet on intimate structure of the liver 204
Liebig, J., on determination of urea 210
Lungs, Dr. F. W. Lewis, cases of obscure diseases of the 257
Littell, Dr., on electricity as a cause of disease 385
Littell, abstract of quarterly report of Will's hospital 513
Lungs, heart and aorta, Dr. W. Walshe's practical treatment on, bibl. notice 608, 665

M

Malformation, Skinner's case of singular fœtal 461
Malignant pustule, Dr. E. D. Ayres on 633
Mason, Dr. J. K., case of placenta previa 199
Mason, Dr. J. K., case of rupture of the uterus, &c. 577
Meigs, Dr. J. F., case of subcutaneous emphysema 7
Meigs, Dr. C. D., treatise on woman, her diseases, &c , bibl. notice 509
Mussey, Dr. R. P., on aneurismal tumors of the ear, both carotid tied 60
Mortality of Philadelphia for Oct. Nov. and Dec. 77
" for Jan. Feb, and March 272
" for April, May and June 465
" for July, Aug. and Sept. 651
Meteorological observations for November, 1853 64
" for December 157
" for January 192
" for February 256
" for March 320
" for April 384
" for May 448
" for June 512
" for July 576
" for August 640
" for September 704
" for October 758
" for the year 1853 128
Medicinal agents not in general use, practical remarks on 125
Medical Society of County of Philadelphia, election of delegates 184
Miller, Dr. Joseph, on mild treatment of scarlatina 187
Monstrosity, Dr. D. M. Hudson's case of 326
McGowen, Dr. Daniel J., report of the Hospital at Ningpo, bib. notice 338
Materia Medica and Therapeutics, Carson's edition of Pereira bib. notice 552
Merinar, Dr. W. H., two cases of successful cesarean section 587
Microscopical anatomy, Prof. Kolliker's manual of human, Da Costa's edition, bib. notice 617
Morris, Dr. C., on death from an acute attack of gout 641
Menorrhagia, by E. Rigby, M.D. 686

N

Natural Sciences, pleasures and advantages of 437
Neill, Dr. John, on use of adhesive plaster in fracture of patella 1
" case of pulsating tumor of the occiput 65
" case of gun-shot wound of the intestine 161
" case of removal of fungus of the upper jaw 195
" hospital cases 449
" gastronomy for rupture of the Uterus 577

Ningpo, report of the hospital for 1852, by Dr. McGowen, bib. notice 338

O

Obstetrics, Spanish 474
Owen, Prof., on the unity and antiquity of the human race 439
" principal forms of the skeleton, &c., bib. notice 677

P

Pancoast, appointment of Prof. Joseph 239
Patterson, Dr. H. S., on treatment of ulcers 139
Patella, use of adhesive plaster in fracture of 1
Prize essay by W. L. Atlee, M.D. 27
" by Samuel G. Armor, M.D. 40
Pulsating tumor of the occiput, Dr. Neill's case of 65
Piggott, Dr. A. S., treatise on chemistry and metallurgy, bib. notice 108
Philadelphia, officers of County Medical Society of 112
Peritonitis, simulating perforation 120
Phthisis, Cleveland's case of, with pneumo-thorax and empyema in an infant 190
Placenta previa, Dr. J. K. Mason's case of 199
Pneumonia, by Dr. R. La Roche, bib. notice 220, 282
Pneumonia, Dr. O. C. Gibbs' case of chronic 265
Pemphigus, report of eighteen cases of 317
Peritonitis, case of tubercular, by Jas. Darrach, M.D. 321
Proceedings of the American Medical Association at St. Louis, 1854 361
Proceedings of the Medical Society of the State of Pennsylvania 422
Preservation of anatomical specimens, Dr. Brinton's method 398
Pennsylvania College, notice of medical department of 431
Peacock, Dr., on malformation of the heart 440
Pepper, Dr. Wm., on treatment of intermittent by quinidine 523
Pereira's Materia Medica, &c., edited by J. Carson, M.D., bib. notice 552
Pleurisy, Dr. A. Flint's clinical report on chronic, bib. notice 563
Phlebitis, traumatic 572
Physiology, Carpenter's principles of comparative, bib. notice 675

Q

Quadruple birth, case of, by S. Kennerly, M.D. 693
Quain, Richard, F.R.S., on diseases of the rectum, bib. notice 179
Quinidine in intermittent fever, by Dr. George L. Upshur 740

R

Radcliffe, Dr. Chas. B., on epilepsy, &c. 500
Ramsbotham, Dr. F. H., cases of labor terminated by forceps 252
Ranking's Half-Yearly Abstract, No. 18, bib. notice 238
Resignation of Dr. H. L. Thomas 43
Report on surgery, by J. B. Flint, M.D., bib. notice of 38
Rectum, diseases of, by R. Quain, F.R.S., bib. notice 179
Richardson, Dr. T. G.'s, elements of human anatomy, bib. notice 237
Rheumatism, rheumatic gout, &c., by Dr. H. W. Fuller, bib. notice 401
Retroversion and retro-flexion of the uterus 447
Rupture of the uterus, gastrotomy performed by Dr. John Neill, Dr. Mason's case of 577
Rupture of the uterus, gastrotomy performed by Dr. J. T. Gilman 694
Rigby, Dr. Edward, on menorrhagia 686

S

Salt, common use of in intermittent fever 241
Sargent, Dr. F. W., notes of a few cases of cholera 529

Scarlatina, Dr. J. Miller's mild treatment of 187
Schröder and Von Dusch on filtration of the air in connection with fermentation and putrefaction 327
Soden, John, case of hæmorrhage from inversion of the uterus 45
Spleen, removal of 59
Simpson, Dr. Jas. T. ,treatise on homœopathy, bib. notice 165
Stokes, Prof. Wm., on diseases of the heart and the aorta, bib. notice 181
Siebold and Stannius comparative anatomy, bib. notice 308
Shaw, Dr. Thos. W., case of cyanosis 459
Skinner, Dr. N. C., case of fœtal malformation 461
Science and art of surgery, by John Erichsen, Prof., bib. notice 477
Stricture of the urethra, Henry Thompson's prize essay on, bib. notice 558
Swift, Dr. H. S., on exhaustion from effects of heat 567
Skoda, Dr., treatise on auscultation, &c., bib. notice 608, 665
Skin, Dr. E. Wilson, on healthy, bib. notice 628
Skeleton and teeth, principal forms of, bib. notice 677
Stillé, Dr. M., on the psychical effects of ether inhalation 730

T

Text-book of anatomy, &c., by W. R. Handy, M.D., bib. notice of 36
Tetanus, caused by arnica, by L. Turck, M.D. 188
Thompson, Dr. Theophilus, clinical lectures on consumption, bib. notice 410
Thompson, Henry, prize essay on stricture of the urethra, bib. notice 558
Therial, Dr., on peritonitis, simulating perforation 120
Tubercular matter, S. Lewis, M.D., on connection of, with inflammation 145
Turck, Dr. Leopold, on tetanus, caused by arnica 188
Tuberculosis, pulmonary, Dr. J. H. Bennett on pathology and treatment of, bib. notice 235
Transactions of the American Medical Association, notice of 313, 349, 418
Types of mankind, bib. notice 343
Therapeutics, short notice of hospital 444
Thomas, Dr., universal formulary, bib. notice 510
Trousseau, Prof, on constipation 571
Thomson, Dr. Thomas, on suppression of hæmorrhage in cancer uteri 639
Tumors, phantom or simulated 681

U

Ulcers of the leg, Dr. H. S. Patterson on treatment of 139
Unity and antiquity of the human race, Prof. Owen's opinions on 439
Use and abuse of alcoholic liquors, by W. B. Carpenter, M.D., bib. notice of 40
Urea, on some of the compounds of, and its determination, by J. Liebig 210
Uterine tumors, Dr. Ashwell on diminution and disappearance of 247
Uterus, Bennett's treatise on inflammation of, bib. notice 356
" retro-version and retroflexion of 447
Upshur, Dr. G. L., on quinidine in intermittent fever 740

V

Venereal disease, A. Vidal, (De Cassis) treatise on, bib. notice 302
Vesico-vaginal fistula, Dr. G. Buck's case of successful operation for 697
Virchow, R., existence of cellulose in the brain 267, 333
Vidal (De Cassis) treatise on venereal disease, bib. notice 302

W

Walshe, Dr. W. H., treatise on diseases of the lungs, &c., bib. notice 608, 665

Wagner, Dr. R., on elementary organization of the brain 601
Water, effects of clyters of pure 249
Wythes, Dr. Joseph H., microscopic examination of a case of Bright's disease 72
Woman, her diseases and remedies, by C. D. Meigs, M.D., bib. notice 509
Wills Hospital, Dr. Littell's abstract of quarterly report 513
Wood and Bache's Dispensatory, 10th edition, bib. notice 561
Wilson, Dr. Erasmus, treatise on healthy skin, bib. notice 628

Z

Zinc, iodide of, employment of 59

